# Influence of synthesis parameters on the structural formation of mayenite via the citrate sol-gel method

**DOI:** 10.55730/1300-0527.3746

**Published:** 2025-07-02

**Authors:** Büşra ERYILDIRIM, Nuray OKTAR, Doruk DOĞU

**Affiliations:** 1 Department of Chemical Engineering, Graduate School of Natural and Applied Sciences, Gazi University, Ankara, Turkiye; 2 Department of Chemical Engineering, Faculty of Engineering, Gazi University, Ankara, Turkiye; 3 Department of Metallurgical and Materials Engineering, School of Engineering, Atılım University, Ankara, Turkiye

**Keywords:** Mayenite, sol-gel, calcination temperature, pH, metal salt to citric acid ratio

## Abstract

Mayenite (Ca_12_Al_14_O_33_) has remarkable properties such as high oxygen mobility, ionic conductivity, and catalytic activity. It has many different applications, including oxide-conducting electrolytes, fluorescent lamps, moisture sensors, hydrogen-permeable membranes, oxygen pumps, hydrogen storage, and catalysis. However, pure and homogeneous mayenite synthesis parameters have not yet been fully explored. This study examines the effect of synthesis parameters including metal salt (MS) to citric acid (CA) molar ratios (1:1 and 1:2), pH (0.4–2), and calcination temperature (900–1200 °C) in citrate sol-gel method on the crystal structure of mayenite. Synthesized materials were examined by thermogravimetric (TG), X-ray diffraction (XRD), X-ray photoelectron spectroscopy (XPS), Raman spectroscopy, N_2_ adsorption-desorption, scanning electron microscopy (SEM), inductively coupled plasma-optical emission spectrometry (ICP-OES), Fourier-transform infrared spectroscopy (FTIR), and pyridine adsorbed diffuse reflectance Fourier-transformed infrared spectroscopy (DRIFTS) analyses. The results show that all 3 parameters contribute to the mayenite phase formation and different impurity phases can be observed depending on the synthesis parameters. With no pH adjustment and an MS to CA ratio of 1, other phases of calcium aluminate mostly form. Mayenite becomes the main phase by doubling the CA amount. Besides CA, pH is also an important factor in mayenite synthesis. When the pH was adjusted to 2 with the MS to CA ratio at 1:1, mayenite was formed as the main phase, but other phases of calcium aluminate were also observed in the structure. XRD results show that all parameters studied influence the crystal structure of the final material, including the calcination temperature. This study shows that pure mayenite can be synthesized with a calcination temperature of 1200 °C, at a pH of 2, and the MS to CA molar ratio of 1:2.

## 1. Introduction

Mayenite (Ca_12_Al_14_O_33_) is a remarkable material that is used in many technological applications such as oxide-conducting electrolytes, fluorescent lamps, moisture sensors, hydrogen-permeable membranes, oxygen pumps, hydrogen storage, H_2_ production from photocatalytic water splitting, and catalytic reactions. This is due to the high oxygen mobility, ionic conductivity, and catalytic properties of mayenite [1–6]. Mayenite is an electrical insulator with a band gap of approximately 7 eV. It can be transformed into an electroactive functional material at low temperatures, such as a metallic conductor with a low work function but high superconductivity and chemical inertness [3]. These extraordinary properties can be attributed to its crystal structure. The crystal structure of mayenite is similar to that of zeolites and clathrate phases of ice, but the positively charged (4+) framework of the unit cell of mayenite is not the same. Mayenite has a crystal structure that can be defined as a nanoporous cubic structure with a unit cell edge of 11.98 Å and 2 Z (formula units) [7–9]. In simple terms, the structure is a framework of AlO_4_ tetrahedra attached to the corner by connections bridged by Ca. The Ca-Al-O framework forms a total of 12 interconnected cages. This framework comprises 32 of the 33 oxygen anions; the extra one is distributed randomly in 1/6 of the large cages within the framework [10]. These oxygen ions, known as extra framework oxygen ions, are loosely bound to the lattice. Six cage-wall Ca^2+^ ions encircle each free oxygen atom with an interionic spacing 1.5× greater than that of the free oxygen in the CaO case. This spacing confirms that oxygen ions are loosely bonded in cages since it is twice the O^2−^ ionic radii (0.24 nm) [3]. These extra framework oxygen ions lead to high oxygen mobility and ionic conductivity. By replacing them with other anions such as OH^−^, N^3−^, Au, Cl^−^, and F^−^, various physical and chemical properties can be achieved, increasing the applications of mayenite [5,10–14].

Mayenite is a mineral that can be found in nature. To obtain pure mayenite in large quantities, it can be synthesized artificially by different methods. The solid-state synthesis route is a simple and conventional method for mayenite synthesis; however, it requires long reaction times and high temperatures (above 1200 °C) [14–21]. Sol-gel synthesis is a promising alternative method for mayenite synthesis because homogeneous and pure mayenite can be obtained at lower temperatures than the solid-state route, and the morphology of the synthesized materials can be controlled and modified easily [22]. Materials with well-defined and homogeneous crystal morphologies can be obtained by sol-gel synthesis [23–25]. In addition, sol-gel synthesis enables doping of the material structure with different ions or metals [2,26].

In the literature, the sol-gel method has been used in the synthesis of nano/meso particles, ceramic-added geopolymers, and nanocomposites. The effects of synthesis parameters on the structure have also been investigated [27–30]. The addition of ceramic additives to geopolymer and nanocomposite systems leads to significant improvements in the thermal, mechanical, and chemical properties of the materials [31]. The unique properties of mayenite, such as high temperature resistance and high electrical conductivity can be used to improve the properties of geopolymers and nanocomposites. Composites containing mayenite and graphene oxide have multifunctional properties, including optical, electrochemical, dielectric, and mechanical properties suitable for high-performance multifunctional applications such as smart cement materials [32].

Although mayenite has many uses, indepth examination of the synthesis parameters is limited. Most of the literature reports on the different uses of mayenite and the impurities found in the structure. Moreover, all synthesis parameters are not clearly defined in these publications. The sol-gel method for mayenite synthesis generally consists of the following steps: hydrolysis of the precursor, condensation, gelling, aging, drying, and calcination. Each of these steps is very important to produce the desired final material. In the sol-gel method, the final structure of the synthesized material is significantly affected by the nature and concentration of precursors, the type of solvent and acidity of the medium, the concentration of each species in the solvent, aging time and temperature, and calcination time and temperature. During the synthesis of mayenite by the sol-gel method, these parameters are very important for the formation of the desired structure because calcium oxide (CaO), aluminum oxide (Al_2_O_3_), and other mixed oxide phases containing aluminum and calcium (Ca_3_Al_2_O_6_, CaAl_2_O_4,_ CaAl_4_O_7_, Ca_5_Al_6_O_14,_ etc.) can also form depending on the synthesis parameters. The ratio of starting materials for mayenite synthesis is fixed at a Ca to Al molar ratio of 12:14. Due to their low cost, nitrate forms of metals were chosen as starting materials, and deionized water was used as a solvent. Citric acid, tartaric acid, glycolic acid, and oxalic acid are popular chelating agents used in the sol-gel process of material synthesis. Citric acid was selected as the chelating agent for this investigation because it is readily available, affordable, and an effective and extensively utilized chelating agent in the sol-gel process [33]. In material synthesis studies using the citrate sol-gel process, the molar ratio of metal salt (MS) to citric acid (CA) is an important parameter affecting the gel formation rate and homogeneity [34]. In addition, some studies have reported that changing the pH of the solution with ammonia or ethylenediamine increases the binding of the cation to citrate. In this regard, the stability and homogeneity of metal citrate solutions largely depend on solution pH [33,35]. Therefore, it was decided to adjust the pH with ethylenediamine and study the effect of the MS to CA molar ratio to optimize the formation of stable and homogeneous metal citrate species. In the CaO-Al_2_O_3_ phase diagram, mayenite forms at 900 °C and above, and other phases of calcium aluminate can also be found in the structure at these temperatures [36]. To obtain pure mayenite, the effect of calcination temperature on the final structure was also investigated. In summary, the effect of MS to CA molar ratio, pH, and calcination temperature were examined in this study to synthesize the pure mayenite structure. The crystal structures of the materials synthesized with different parameters were determined by X-ray diffraction (XRD) analysis. Thermogravimetric (TG), Fourier-transform infrared spectroscopy (FTIR), pyridine adsorbed diffuse reflectance Fourier-transformed infrared spectroscopy (DRIFTS), N_2_ adsorption-desorption analysis, scanning electron microscopy (SEM) analysis, laser Raman spectroscopy, X-ray photoelectron spectroscopy (XPS), and inductively coupled plasma-optical emission spectrometry (ICP-OES) analysis were also performed. This study clearly presents the mayenite synthesis parameters and provides the optimum synthesis route by determining the effect of the parameters on the final structure. In most mayenite synthesis studies, the synthesis parameters are not specified, and the resulting material may contain impurities. In addition, there are no studies examining the effects of the MS to CA molar ratio, pH, and calcination temperature together with detailed analysis techniques. In this respect, the findings of this study will be a valuable resource for other researchers who need to synthesize pure mayenite.

## 2. Materials and methods

### 2.1. Preparation of materials

**Figure 1:**
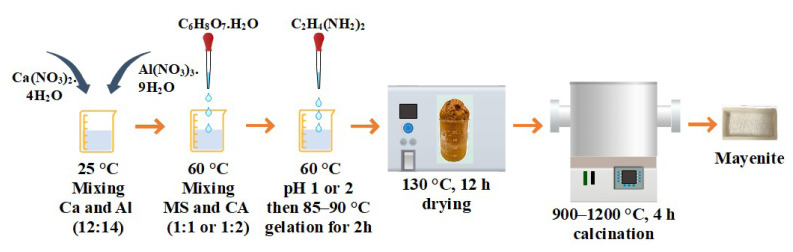
A schematic diagram of the citrate sol-gel method for mayenite synthesis.

Mayenite materials were synthesized by a citrate sol-gel procedure [35] using the following steps: preparation of the MS solution, gel formation, drying, and calcination. In this procedure, the starting materials calcium nitrate tetrahydrate (Ca(NO_3_)_2_.4H_2_O_,_ Merck, Darmstadt, Germany) and aluminum nitrate tetrahydrate (Al(NO_3_)_3_.9H_2_O, Merck, Darmstadt, Germany) were dissolved in deionized water (50 mL) at room temperature to obtain a Ca to Al molar ratio of 12:14. The total concentration of the resulting solution was 1 M. The solution was then heated to 60 °C under continuous stirring. The CA solution used as a chelating agent was prepared in another beaker. Enough CA (C_6_H_8_O_7_, Sigma-Aldrich, St. Louis, MO, USA) was added to deionized water (50 mL) to obtain a total MS to CA molar ratio of 1:1 or 1:2. The CA solution was added to the MS solution under continuous stirring at 60 °C in a dropwise manor. At this stage, the desired pH (1 or 2) of the solution was adjusted with ethylenediamine (C_2_H_4_(NH_2_)_2_, Sigma-Aldrich, St. Louis, MO, USA). Synthesis without pH adjustment was also tested in this study. After pH adjustment, the temperature was increased to 85–90 °C and kept at this temperature under continuous stirring until a viscous gel was formed (after about 2h). The obtained gel was then dried in the oven at 130 °C for 12 h to obtain a cake-like structure. This cake-like structure was pulverized to obtain a powder. Finally, the powder was calcined under a flow of dry air. All samples were heated to the calcination temperature with a ramp rate of 5 °C/min from room temperature and calcined in a tube furnace at the desired calcination temperature (900, 1000, 1100, and 1200 °C) for 4 h. A schematic representation of the synthesis of mayenite by the citrate sol-gel method is given in Figure 1.

### 2.2. Characterization of the synthesized materials

TG analyses of the synthesized materials were carried out at the Central Laboratory of Ankara Yıldırım Beyazıt University. Analyses were performed on a HITACHI STA 7300 instrument (Tokyo, Japan). TG analyses were carried out at temperatures between 25 and 1200 °C with a heating rate of 5 °C/min under dry air flow.

To analyze the crystal structure of the synthesized materials, XRD analysis was conducted using a Bruker D2 Phaser diffractometer (Billerica, MA, USA) with a Cu, K_α_ (λ = 0.15406 nm) radiation source at the Metal Forming Center of Excellence (MFCE) in Atılım University. XRD analyses were performed between the 2θ range of 10°–90° with a scanning speed of 2°/min. Crystal sizes of the synthesized materials were calculated using the Scherrer equation (L = Kλ/βcosθ). In this equation, L, the crystal size, is a measure of the dimension of the particle in the direction perpendicular to the reflecting plane; K is the shape factor depending on the sphericity of the crystal that is commonly taken as 0.89 as in this study; λ is the X-ray wavelength (0.15406 nm); β is the full width at half maxima in radian of the strongest peak (2θ approximately 18° is taken); and θ is the Bragg angle, the angle between the incident beam and the reflecting plane that leads to constructive interference of X-ray beams and hence a peak is observed [37].

FTIR and pyridine adsorbed DRIFTS analyses were performed using a JASCO Model FT/IR-4700 instrument (Tokyo, Japan). Laser Raman spectroscopy analyses were performed at room temperature using a JASCO NRS4500 Raman spectrometer (Tokyo, Japan) with a 532 nm ion laser.

XPS analysis using a PHI 5000 VersaProbe (ULVAC-PHI, Kanagawa, Japan) instrument was performed to define the surface chemistry of the materials. Data was collected for C 1s, O 1s, Al 2p, and Ca 2p regions, and peak correction was made for the C 1s peak at 284.5 eV. XPSPEAK41 software was used for curve fitting.

N_2_ adsorption-desorption analysis was performed using a Micromeritics Tristar II 3020 device (Norcross, GA, USA) at 77 K within P/P_o_ values from 10^−5^ to 0.99 to obtain the surface areas of synthesized materials. Synthesized materials were degassed under a vacuum at 250 °C for 2 h before analysis.

SEM analysis was performed with the HITACHI SU5000 field emission high-resolution scanning electron microscope (Tokyo, Japan) to assign the morphology of the synthesized materials. Metal ratios of the synthesized materials were assigned by ICP-OES analysis with a PerkinElmer Optima 4300DV instrument (Waltham, MA, USA).

## 3. Results and discussion

### 3.1. Results of synthesized materials with a MS to CA ratio of 1

The synthesis studies were first carried out by keeping the MS to CA molar ratio at 1 with no pH adjustment (pH of 0.6). After that, the effect of pH was also tested by adjusting the pH to 2 by adding ethylenediamine to the solution.

**Figure 2:**
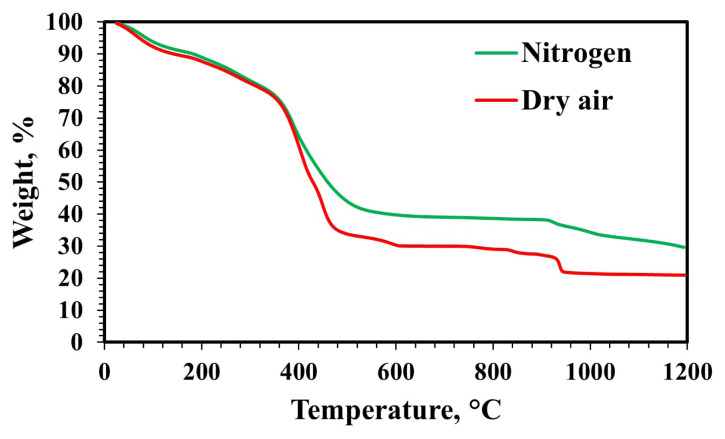
TG analysis results of the material synthesized without pH adjustment (MS:CA 1:1, pH of 0.6).

**Figure 3:**
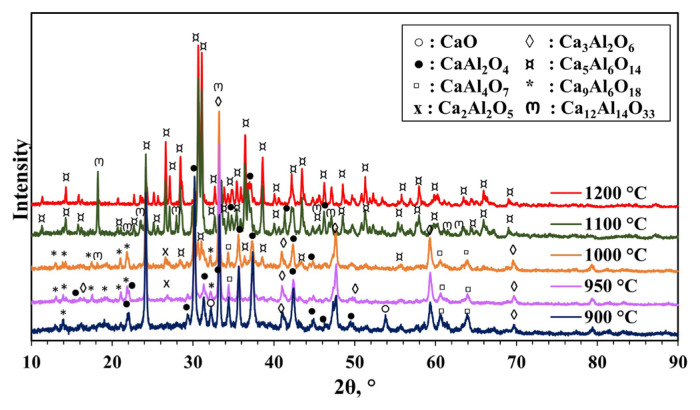
XRD patterns of materials synthesized without pH adjustment and calcined at different temperatures (900–1200 °C) for 4 h (MS:CA 1:1, pH of 0.6).

TG analyses were conducted between 25–1200 °C with a heating rate of 5 °C/min under dry airflow and nitrogen flow (Figure 2). TG analysis showed weight losses of up to 200 °C due to water removal from the material structure. Weight losses between 250–950 °C occurred due to nitrate decomposition and the combustion of other organic compounds [2,12,38]. The mass of the material became stable in a dry air atmosphere at approximately 950 °C. Below 950 °C, all metal nitrate compounds are completely decomposed, and above this temperature, solid-solid reactions occur between calcium oxide and alumina. According to the CaO-Al_2_O_3_ phase diagram, the possible phases above 1000 °C are Ca_12_Al_14_O_33_, Ca_3_Al_2_O_6_, CaAl_2_O_4_, and CaAl_4_O_7_ [36]. In the XRD analysis results, these phases were present in the structure (Figure 3). Mass loss continued in the nitrogen atmosphere after 950 °C. In addition, the final powder after TG analysis under nitrogen flow was observed to be black in color, indicating carbon compounds remain in the structure. Therefore, synthesis studies were continued in a dry air atmosphere.

XRD patterns (2θ range of 10°–90°) of materials synthesized without pH adjustment at different calcination temperatures from 900 to 1200 °C for 4 h are shown in Figure 3. Characteristic peaks of mayenite were not observed in the XRD patterns of the synthesized materials at 900 and 950 °C. Ca_3_Al_2_O_6_ (COD 9014359) [39] and CaAl_2_O_4_ (COD 2002888) [40] phases, which are the usual impurities formed during mayenite synthesis, were mainly formed. XRD pattern of mayenite presents the characteristic peaks of mayenite around 2θ values of 18.1°, 30°, 33.4°, 36.7°, 41.2°, 46.7°, 55.2°, and 57.4° (COD 2102955) [41,42]. At 1000 °C, a characteristic peak of mayenite (2θ approximately 18°) was observed with low peak intensity. This low peak intensity indicates that mayenite is not the main structure in this synthesized material. At 1100 °C, mainly Ca_5_Al_6_O_14_ (COD 2106611) [43] was observed, and the peak intensities of this phase and the mayenite phase increased. At 1200 °C, the Ca_5_Al_6_O_14_ phase was formed and the mayenite phase disappeared at this temperature.

The phases and percent crystallinity of the synthesized material obtained at different calcination temperatures are given in Table 1. Phases and crystallinities of the synthesized materials were determined with the DIFFRAC.EVA program, the Bruker D2 Phaser diffractometer software. By comparing the signal to noise ratio in these diffractograms, one can observe that the crystallinity increases with increasing calcination temperature, as expected.

**Figure 4:**
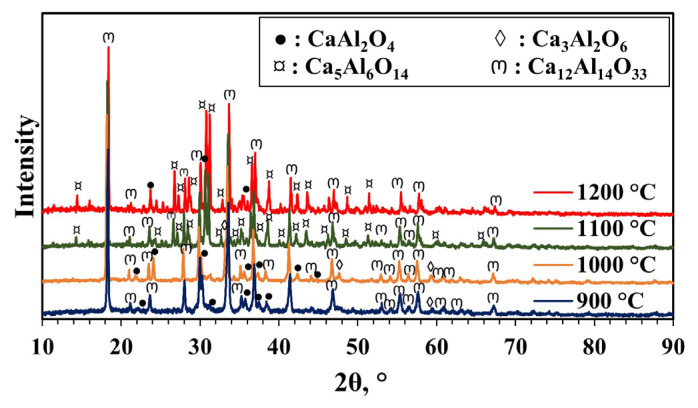
XRD patterns of materials synthesized with pH adjustment and calcined at different temperatures (900–1200 °C) for 4 h (MS:CA 1:1, pH of 2).

To examine the effect of pH, it was adjusted to 2 while the MS to CA ratio was kept at 1. Contrary to the material without pH adjustment, the mayenite phase was formed at all temperatures after calcination was carried out between 900 and 1200 °C. Impurities (Ca_5_Al_6_O_14_, Ca_3_Al_2_O_6,_ and CaAl_2_O_4_) formed in addition to the mayenite main phase according to the XRD patterns (Figure 4 and Table 1). Intiso et al. [44] also detected Ca_3_Al_2_O_6_ and CaAl_2_O_4_ phases in the mayenite. They used the citrate sol-gel method for synthesis. The crystal sizes of the synthesized mayenite phases at different calcination temperatures were calculated using the Scherrer equation. The crystal size of the material was 79.6 nm and was not affected by changing the calcination temperature at the pH value of 2. Crystallinity increased slightly with increasing calcination temperature.

### 3.2. Results of synthesized materials with a MS to CA ratio of 1:2

The next set of experiments focused on synthesis studies done at a MS to CA molar ratio of 1:2. Synthesis studies were carried out without pH adjustment (pH: 0.43) and by adjusting the pH level to 1 and 2 using ethylenediamine.

**Figure 5:**
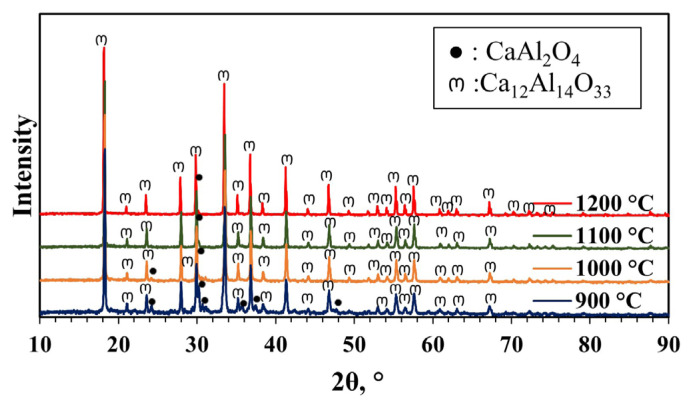
XRD patterns of materials synthesized without pH adjustment and calcined at different temperatures (900–1200 °C) for 4 h (MS:CA 1:2, pH of 0.43).

The XRD patterns of the materials synthesized with a pH of 0.43 are given in Figure 5. At 900 °C, there was a CaAl_2_O_4_ impurity phase in the material structure in addition to the mayenite phase. As the calcination temperature increased, the formation of CaAl_2_O_4_ phase decreased. Additionally, crystallinity increased with increasing the calcination temperature (Table 1). When the molar amount of CA was doubled (Figure 5), the mayenite phase was formed as the main phase at all temperatures compared to the material without pH adjustment (Figure 3). This result shows how important the amount of CA in the synthesis solution is for the final structure.

**Figure 6:**
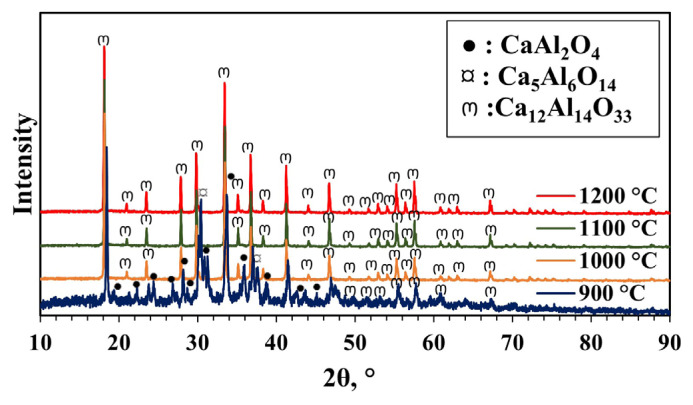
XRD patterns of materials synthesized with pH adjustment and calcined at different temperatures (900–1200 °C) for 4 h (MS:CA 1:2, pH of 1).

The XRD patterns of the materials synthesized with a pH of 1 are given in Figure 6. Similar to the results obtained in the material with a pH of 0.43, there was CaAl_2_O_4_ phase in addition to the mayenite phase in the material structure at 900 °C, and an almost pure mayenite phase was obtained with calcinations done above 1000 °C (Table 1). Additionally, a small amount of Ca_5_Al_6_O_14_ phase was observed at 900 °C. Changing the calcination temperature did not cause a change in the crystal size (79.6 nm) of the material and the crystallinity of the material increased with increasing calcination temperature (Table 1).

**Figure 7:**
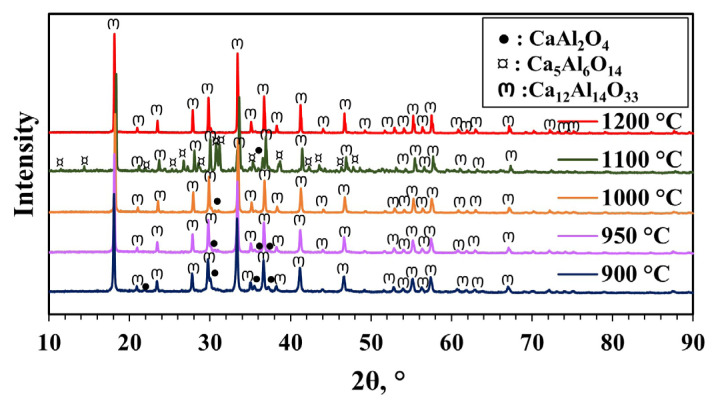
XRD patterns of materials synthesized with pH adjustment and calcined at different temperatures (900–1200 °C) for 4 h (MS:CA 1:2, pH of 2).

The XRD patterns of the materials synthesized with a pH of 2 are given in Figure 7. The mayenite phase formed at 900, 950, 1000, and 1200 °C, along with a trace amount of the CaAl_2_O_4_ phase, which is a possible impurity during synthesis. At 900 °C, the crystal structure contained much less impurities compared to the materials synthesized with a pH of 0.43 and 1. Unlike other pH levels, the mayenite phase again formed at 1100 °C, but the side phases Ca_5_Al_6_O_14_ and CaAl_2_O_4_, which are the usual impurities formed during the mayenite synthesis, were also formed. Sanchez et al. [4] also reported the formation of the Ca_5_Al_6_O_14_ impurity phase in the synthesis of mayenite at a calcination temperature of 1100 °C. When the temperature was increased to 1200 °C, the pure mayenite phase was present without indication of any impurities. Similar to the synthesized materials with other parameters, the crystal size (79.6 nm) of the material was not affected by the change in calcination temperature. Additionally, the crystallinity of the material increased with increasing calcination temperature (except 1100 °C) (Table 1).

**Figure 8:**
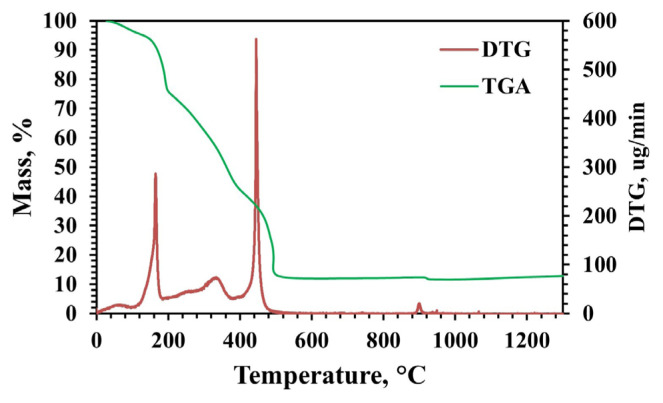
TG analysis results of material synthesized with pH adjustment (MS:CA 1:2, pH of 2). (TGA: thermogravimetric analysis and DTG: derivative thermogravimetry)

TG, N_2_ adsorption-desorption, SEM, FTIR, Raman, XPS, pyridine adsorbed DRIFTS, and ICP-OES analyses were carried out with an MS to CA molar ratio of 1:2 and pH 2, where the pure mayenite phase was obtained. The effects of temperature on the structure were investigated by applying the analyses to samples at different calcination temperatures (900–1200 °C). TG analysis of the material with an MS to CA molar ratio of 1:2 and a pH of 2 was carried out at 30–1300 °C with a heating rate of 5 °C/min under dry air flow. Figure 8 displays the findings of the TG analysis investigation of precalcination powder obtained in mayenite synthesis. Weight loss up to 160 °C was caused by water evaporation since organic species are stable below this temperature. Nitrates burn with the remaining simple CA at temperatures between 160 and 275 °C, causing a significant weight loss. Metal citrate complexes break down into metal oxides between 275 and 380 °C, and all organic substances burn through oxidative degradation at temperatures exceeding 380 °C [35]. According to derivative thermogravimetry (DTG), the mass loss that occurs at approximately 373 °C is due to the decomposition of the aluminum nitrate salt into alumina (2Al(NO_3_)_3_ → Al_2_O_3_ + NO_2_ + O_2_) [2]. At temperatures above 50 °C, CA was used as a chelating agent that reacts with calcium nitrate to form calcium citrate (3Ca(NO_3_)_2_.4H_2_O + 3C_6_H_8_O_7_ → Ca_3_(C_6_H_5_O_7_)_2_.4H_2_O + 3CO_2_ +7H_2_O + 6NO_2_). At a temperature of approximately 474 °C, calcium citrate decomposes into calcium carbonate (Ca_3_(C_6_H_5_O_7_)_2_ → 3CaCO_3_ + 5H_2_O +9C) [45]. Finally, at approximately 919 °C, the calcium carbonate turns into calcium oxide CaO (CaCO_3_ → CaO + CO_2_) [2,45]. Salasin et al. [35] achieved a similar TG analysis profile in the synthesis of mayenite using the citrate sol-gel method.

**Figure 9:**
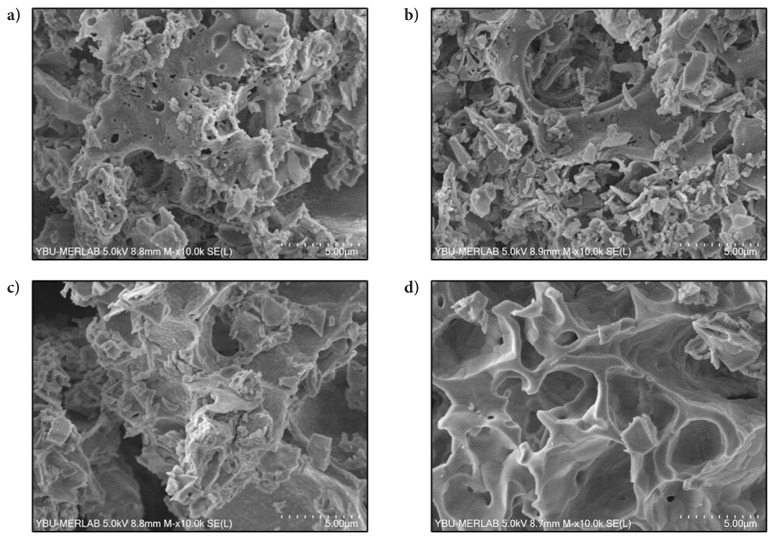
SEM images of materials synthesized with pH adjustment and calcined at different calcination temperatures for 4 h (MS:CA 1:2, pH of 2) (a) 900 °C, (b) 1000 °C, (c) 1100 °C, and (d) 1200 °C.

The morphology of the synthesized materials at 900, 1000, 1100, and 1200 °C were investigated by SEM analysis. The images that are shown in Figure 9 display randomly shaped particles in materials synthesized using different calcination temperatures. The increase in calcination temperature makes the structure more homogeneous and regular. Raab et al. [46] obtained a similar structure in the SEM image of synthesized mayenite at 1200 °C.

N_2_ adsorption-desorption analysis determined the specific surface area of mayenite materials synthesized at different temperatures. The surface areas of the synthesized materials were less than 1 m^2^/g (Table 2). Additionally, there was a decrease in surface area values with increasing temperature. Mironova et al. [47] reported that the specific surface area of the mayenite they synthesized was 0.5 m^2^/g.

**Figure 10:**
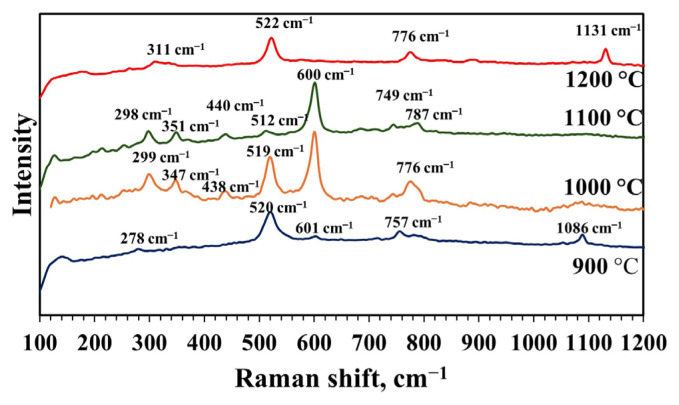
Raman spectra of materials synthesized with pH adjustment and calcined at different calcination temperatures (900–1200 °C) for 4 h (MS:CA 1:2, pH of 2).

Raman spectroscopy was used in addition to XRD to verify the phase identity of synthesized mayenite materials. Raman analyses results of the material synthesized with an MS to CA molar ratio of 1:2, a pH of 2, and calcined at 900, 1000, 1100, and 1200 °C are shown in Figure 10. The mayenite material has several modes in the range of 150–1000 cm^−1^ attributed to Al-O framework vibrations. The most dominant mode of mayenite is around 520 cm^−1^, attributed to the symmetric Al-O-Al vibration of the oxygen bridge between the AlO_4_ tetrahedrons. At approximately 311 cm^−1^, there is an additional weaker and broader line that is thought to be caused by framework vibrations. The vibrations seen in the regions around 776 and 1131 cm^−1^ are attributed to the vibrations of the extra framework O_2_^−^ species [14,48–51]. The material synthesized at 900 °C has 2 bands in the Raman spectrum other than the vibrations of pure mayenite material. The high-frequency band at 1086 cm^−1^ can be associated with the O_2_^−^ structure, and the low-frequency band at 278 cm^−1^ is due to a vibrational group in the Ca and O structure. Reports suggest that the band at 1086 cm^−1^ can be attributed to both CaCO_3_ [50,52] and extra framework oxygen in the structure [53]. XRD results did not indicate CaCO_3_ in the structure (Figure 7); however, this may have been caused by the crystal size of this phase being too small. The Raman spectra of materials synthesized at 1000 and 1100 °C yielded extra bands around 300, 350, and 440 cm^−1^. The weak bands at approximately 300 and 350 cm^−1^ may be associated with the Ca-O bond or framework O [51,54]. The weak band at approximately 440 cm^−1^ might originate from the CaAl_2_O_4_ phase in the structure, and this band is related to the AlO_4_^5−^ vibration [54]. The sharp band seen at 600 cm^−1^ at both temperatures is the characteristic band of the Ca_5_Al_6_O_14_ phase [48,51]. In the XRD pattern of the material synthesized at 1100 °C, an excess of Ca_5_Al_6_O_14_ phase was detected in the structure (Figure 7). This result also supports the XRD analysis result. At 1000 °C, only CaAl_2_O_4_ and Ca_12_Al_14_O_33_ phases were detected in the XRD result. The Ca_5_Al_6_O_14_ phase was not seen in XRD due to the low concentration of the Ca_5_Al_6_O_14_ phase or small crystal sizes. The Raman spectrum obtained from the calcined material at 1200 °C is in accordance with the pure mayenite spectrum. The XRD pattern of synthesized material at this temperature also shows a pure mayenite phase, which supports the Raman analysis result. The main Raman bands and phase interpretations obtained are given in Table 3.

**Figure 11:**
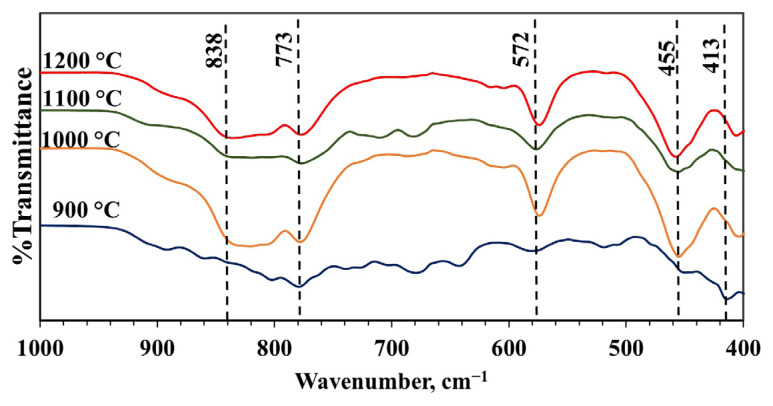
FTIR spectra of materials synthesized with pH adjustment and calcined at different temperatures (900–1200 °C) for 4 h (MS:CA 1:2, pH of 2).

FTIR spectra were used to evaluate the vibration mode of the atomic bonding of material synthesized with an MS to CA molar ratio of 1:2, a pH of 2, and calcined at 900, 1000, 1100, and 1200 °C (Figure 11). The aluminum coordination number, the coordination group state (isolated or condensed), and the vibrational coupling between adjacent units affect the FTIR spectra of inorganic aluminates. Characteristic IR absorbance frequency ranges are 900–700 cm^−l^ for condensed AlO_4_ tetrahedra, 800–650 cm^−l^ for isolated AlO_4_ tetrahedra, 680–500 cm^−l^ for condensed AlO_6_ octahedra, and 530–400 cm^−l^ for isolated AlO_6_ octahedra [55]. The FTIR patterns of calcium aluminate compounds are quite different, but they are characterized by a strong vibration in the region of 900–760 cm^−l^. This vibration should be associated with AlO_4_ tetrahedra or, more specifically, the stretching vibrations of a lattice of interconnected AlO_4_ tetrahedra [55]. The peak at 773 cm^−1^ may also be associated with the free oxygen in the framework structure of mayenite. The 572 and 455 cm^−1^ vibrational bands are related to the bonds of Ca, Al, and O atoms (Ca-O-Al stretching) [32]. Some bending motion of the AlO_4_ lattice may have contributed to the strong absorption observed in the 400–500 cm^−l^ region.

While similar spectra are obtained at temperatures of 1000 °C and above in the spectra of samples synthesized at different calcination temperatures, band vibrations are weaker or absent at 900 °C. Unlike other temperatures, the vibration observed at 900 °C can be attributed to the Ca-O bond [56]. The conclusion that the vibration seen in the Raman spectrum of 1086 cm^−1^ is caused by CaCO_3_ can be supported by this result obtained in the FTIR spectrum. Also, in the TG analysis (Figure 8), CaCO_3_ decomposed into CaO at approximately 919 °C, which may suggest that there is a small amount of CaCO_3_ left in the structure. As a result, mayenite synthesized at 1000 °C and above has AlO_4_-tetrahedral sites, and FTIR spectra show the characteristic peaks for both condensed and isolated AlO_4_ tetrahedral peaks.

**Figure 12:**
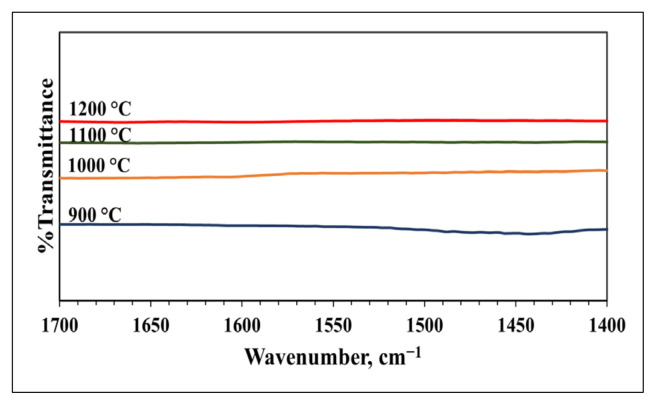
Pyridine adsorbed DRIFTS spectra of materials synthesized with pH adjustment and calcined at different temperatures (900–1200 °C) for 4 h (MS:CA 1:2, pH of 2).

Pyridine adsorbed DRIFTS analysis was performed to determine the surface acidity of materials synthesized at various calcination temperatures. The vibration bands corresponding to pyridine species were observed in the 1400–1650 cm^−1^ region. Materials did not have peaks in the wave number range of 1400–1650 cm^−1^, indicating no acidic sites on the synthesized materials (Figure 12).

**Figure 13:**
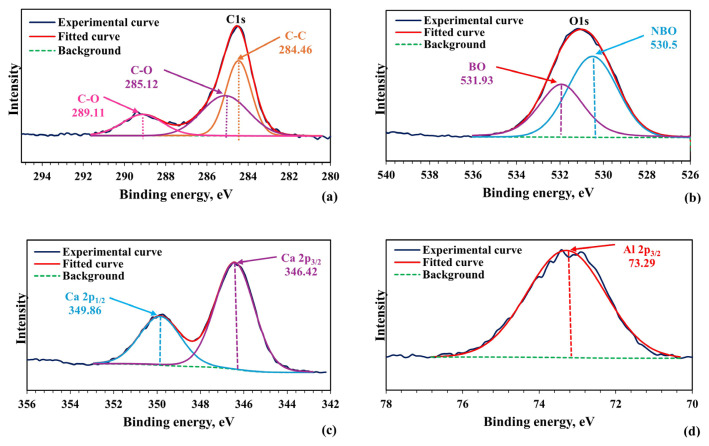
XPS spectra of material synthesized with pH adjustment and calcined at 1200 °C for 4 h (MS:CA 1:2, pH of 2) **(a)** C 1s, **(b)** O 1s, **(c)** Ca 2p and **(d)** Al 2p.

XPS analysis determined the surface properties of material synthesized with an MS to CA molar ratio of 1:2, pH of 2, and calcined at 1200 °C. XPS spectra were collected for the C 1s, O 1s, Al 2p, and Ca 2p regions of the samples (Figure 13). C 1s peak data were first examined for the configuration and confirmation of XPS spectra. The peak that was positioned after fitting was located at 284.46 eV (Figure 13a) [57]. Deconvolution of the O 1s spectrum shows 2 separate contributions, with peaks located at around 530.5 eV and 531.93 eV, respectively (Figure 13b). The high-energy component at 531.93 eV is attributed to the bridging oxygen (BO) atoms, while the low-energy component at 530.5 eV is assigned to nonbridgeable oxygen (NBO) atoms [58]. The Ca 2p XPS spectra show 2 peaks at around 346.42 eV and 349.86 eV, which are related to Ca 2p_3/2_ and Ca 2p_1/2_, respectively (Figure 13c). These 2 peaks result from spin-orbit splitting in the Ca 2p XPS spectra. These findings show that Ca and O are linked, creating CaO [58–60]. The Al 2p XPS spectra have peak locations around 73.29 eV, indicating that the valence state of Al (+3) in Ca_12_Al_14_O_33_ is the same as that of Al_2_O_3_ (Figure 13d) [57–60]. It is concluded that the bonds of Ca-O and Al-O exist in the structure of the mayenite synthesized from the resulting XPS spectra.

ICP-OES analysis was also conducted to verify the Ca and Al ratios of material synthesized with an MS to CA molar ratio of 1:2, a pH of 2, and calcined at 1200 °C. The ICP-OES analysis shows that the material contained 34 ± 1% Ca and 26 ± 1% Al by weight and was synthesized in the desired ratios.

Abbreviations and their definitions used in this paper are given in Table 4.

## 4. Conclusions

This study investigated the effects of MS to CA molar ratio, pH, and calcination temperature on the final structure of mayenite synthesized using the citrate sol-gel method. The crystal structure of synthesized materials with different synthesis parameters was investigated using XRD. With the MS to CA ratio of 1, different calcium aluminate phases were formed in materials without pH adjustment; however, mayenite was not formed except at 1000 and 1100 °C. Even at these temperatures, mayenite is not the main phase obtained. When the pH of the solution was adjusted to 2 and the MS to CA ratio is kept at 1, the mayenite phase formed as the dominant phase in the structure at all calcination temperatures. On the other hand, a significant amount of Ca_5_Al_6_O_14_, Ca_3_Al_2_O_6_, and CaAl_2_O_4_ impurities were also observed in the structure at all temperatures. The crystallinity of the samples increases with increasing calcination temperature, as expected.

This study shows that the MS to CA molar ratio is more important than pH in synthesizing pure mayenite. When the MS to CA ratio was 1:2, mayenite formed as the main phase in the structure at all pH levels and calcination temperatures. With no pH adjustment and the MS to CA ratio of 1:2, there was a CaAl_2_O_4_ impurity phase in the material structure in addition to the mayenite phase at 900 °C. The increase in calcination temperature led to a decrease in this impurity phase and increased crystallinity. With a pH of 1, Ca_5_Al_6_O_14_ was also observed as an impurity in addition to CaAl_2_O_4_ at 900 °C. By increasing the calcination temperature, the crystallinity in the samples increases and impurities decrease. Above 1000 °C, almost pure mayenite was obtained.

With the MS to CA ratio at 1:2 and a pH of 2, CaAl_2_O_4_ was the only impurity phase at calcination temperatures of 900, 950, and 1000 °C, but it was at a lower level than at a pH of 0.43 and 1. XRD results showed that the sample calcined at 1100 °C had a small amount of Ca_5_Al_6_O_14_ impurities in its structure besides CaAl_2_O_4_. When the calcination temperature was increased to 1200 °C, no impurities were observed in the structure and pure mayenite was obtained. Crystallinity increased with increasing calcination temperature, reaching a maximum of 87% at 1200 °C. Mayenite crystal sizes was not affected by the synthesis parameters, and it was calculated as 79.6 nm for all samples containing mayenite. The purity of the mayenite structure formed at 1200 °C with a MS to CA ratio of 1:2 and a pH of 2 was confirmed by Raman, FTIR, and XPS analysis in addition to XRD. Moreover, ICP-OES analysis on this sample showed that it contained 34 ± 1% Ca and 26 ± 1% Al by weight, which is compatible with the amounts in mayenite. N_2_ adsorption-desorption analysis was also performed on samples synthesized with the MS to CA ratio of 1:2 and pH of 2, showing that the surface area decreased from 0.71 to 0.13 m^2^/g as the calcination temperature increased from 900 to 1200 °C, respectively.

In summary, this study establishes the optimal synthesis conditions for mayenite and investigates the impact of various synthesis parameters on its structural properties. The results show that these parameters significantly influence the final crystal structure of mayenite synthesized via the citrate sol-gel method. The most favorable conditions for preparing pure mayenite are an MS to CA molar ratio of 1:2 and calcination temperatures exceeding 1000 °C, with 1200 °C yielding the highest purity. Additionally, pH has less of an effect on the formation of pure mayenite than the MS to CA molar ratio and calcination temperature. The findings of this study offer valuable insights for any research involving mayenite. The synthesis method for pure mayenite presented herein has the potential to significantly improve the performance of various applications, including oxide-conducting electrolytes, fluorescent lamps, moisture sensors, hydrogen-permeable membranes, oxygen pumps, hydrogen storage systems, and catalytic processes.

## Figures and Tables

**Table 1:** Phases identified and crystallinity using X-ray powder diffraction.

MS/CA	1				1/2					
Calcination Temperature, °C	Phases Present		Crystallinity, %		Phases Present			Crystallinity, %		
	pH: 0.6	pH: 2	pH:0.6	pH:2	pH: 0.43	pH: 1	pH: 2	pH: 0.43	pH: 1	pH: 2
900	CaO, CaAl_2_O_4_, CaAl_4_O_7_, Ca_3_Al_2_O_6_, Ca_9_Al_6_O_18_	CaAl_2_O_4_, Ca_3_Al_2_O_6_, Ca_12_Al_14_O_33_	73	76	CaAl_2_O_4,_ Ca_12_Al_14_O_33_	CaAl_2_O_4,_ Ca_5_Al_6_O_14_, Ca_12_Al_14_O_33_	CaAl_2_O_4,_ Ca_12_Al_14_O_33_	78	74	83
950	CaAl_2_O_4_, CaAl_4_O_7_, Ca_2_Al_2_O_5_, Ca_3_Al_2_O_6_, Ca_9_Al_6_O_18_	-	76	-	-	-	CaAl_2_O_4,_ Ca_12_Al_14_O_33_	-	-	85
1000	CaAl_2_O_4_, CaAl_4_O_7_, Ca_2_Al_2_O_5_, Ca_3_Al_2_O_6_, Ca_5_Al_6_O_14_, Ca_9_Al_6_O_18_, Ca_12_Al_14_O_33_	CaAl_2_O_4_, Ca_3_Al_2_O_6_, Ca_12_Al_14_O_33_	78	79	CaAl_2_O_4,_ Ca_12_Al_14_O_33_	CaAl_2_O_4,_ Ca_12_Al_14_O_33_	CaAl_2_O_4,_ Ca_12_Al_14_O_33_	84	82	84
1100	CaAl_2_O_4_, Ca_5_Al_6_O_14_, Ca_9_Al_6_O_18_, Ca_12_Al_14_O_33_	Ca_5_Al_6_O_14_, Ca_12_Al_14_O_33_	80	80	CaAl_2_O_4,_ Ca_12_Al_14_O_33_	CaAl_2_O_4,_ Ca_12_Al_14_O_33_	CaAl_2_O_4,_ Ca_5_Al_6_O_14_, Ca_12_Al_14_O_33_	84	85	82
1200	Ca_5_Al_6_O_14_	CaAl_2_O_4_, Ca_5_Al_6_O_14_, Ca_12_Al_14_O_33_	81	79	CaAl_2_O_4,_ Ca_12_Al_14_O_33_	CaAl_2_O_4,_ Ca_12_Al_14_O_33_	Ca_12_Al_14_O_33_	86	87	87

**Table 2:** N_2_ adsorption-desorption results of materials synthesized with pH adjustment and calcined at different calcination temperatures for 4 h (MS:CA 1:2, pH of 2).

Calcination Temperature, °C	Surface Area, m^2^/g
900	0.71
1000	0.52
1100	0.48
1200	0.13

**Table 3:** The main Raman bands and their identified phases.

Raman Shift, cm^−1^	Phase Identity	Assignment	Assignment references
278	CaCO_3_	Framework Ca	[52]
299, 311	Ca_12_Al_14_O_33_	Framework O	[14,51]
347, 351	Ca_12_Al_14_O_33_	Framework O	[14,53]
438, 440	CaAl_2_O_4_	Framework Al	[54]
512,519,520,522	Ca_12_Al_14_O_33_	Framework O and Al	[14,48–51]
600, 601	Ca_5_Al_6_O_14_	Framework O and Al	[14,48,51]
749, 757	Ca_12_Al_14_O_33_	Framework O, Al	[53]
776, 787	Ca_12_Al_14_O_33_	Extra framework O_2_^−^	[14,48–51]
1086	Ca_12_Al_14_O_33_ or CaCO_3_	Extra framework O_2_^−^ or framework Ca and O	[50,52,53]
1131	Ca_12_Al_14_O_33_	Extra framework O_2_^−^	[14,48–51]

**Table 4:** List of abbreviations and acronyms used in the article.

Abbreviation	Definition
CA	Citric acid
cm^−1^	Reciprocal centimeter
DRIFTS	Diffuse reflectance Fourier-transformed infrared spectroscopy
DTG	Derivative thermogravimetry
eV	Electron volt
FTIR	Fourier-transform infrared spectroscopy
ICP-OES	Inductively coupled plasma-optical emission spectrometry
K	Constant in the Scherrer equation
L	Measure of the dimension of the particle in the direction perpendicular to the reflecting plane
MS	Metal salt
nm	Nanometer
SEM	Scanning electron microscopy
TG	Thermogravimetric
XPS	X-ray photoelectron spectroscopy
XRD	X-ray diffraction
β	Full width at half maxima of the strongest peak in the XRD pattern
θ	Angle between the beam and the normal on the reflecting plane
λ	X-ray wavelength
